# A Subunit Vaccine Candidate Composed of Mpox Virus A29L, M1R, A35R, and B6R Elicits Robust Immune Response in Mice

**DOI:** 10.3390/vaccines11091420

**Published:** 2023-08-25

**Authors:** Xuetao Yang, Xidan Yang, Shouwen Du, Congxia Hu, Xiu Yang, Xingyun Wang, Xing Hu, Nino Rcheulishvili, Peng George Wang, Jihui Lin

**Affiliations:** 1 School of Nursing, Southwest Medical University, Luzhou 646000, China; 20210799120005@stu.swmu.edu.cn (X.Y.); 20210799120002@stu.swmu.edu.cn (X.Y.); congxiahu0606@sjtu.edu.cn (C.H.); 20220799120041@stu.swmu.edu.cn (X.Y.); 2 Pengbo Biotechnology Co., Ltd., Shenzhen 518000, China; 3 Department of Infectious Diseases, Shenzhen People’s Hospital (The First Affiliated Hospital, Southern University of Science and Technology), The Second Clinical Medical College of Jinan University, Shenzhen 518020, China; du-guhong@163.com; 4 Department of Pharmacology, School of Medicine, Southern University of Science and Technology, Shenzhen 518000, China; 12032609@mail.sustech.edu.cn (X.W.); 12032620@mail.sustech.edu.cn (X.H.);; 5 Institute of Microbiology, Chinese Academy of Sciences (CAS), Beijing 100101, China

**Keywords:** Mpox virus, subunit vaccine, CpG7909 adjuvant, prokaryotic expression, immune response

## Abstract

With no specific antiviral drugs and preventive vaccines against Mpox virus (MPXV), the epidemic has led to the declaration of a Public Health Emergency of International Concern. As a developmental direction for new vaccines, studies of subunit vaccines based upon MPXV antigen proteins are lacking. In this study, A29L, M1R, A35R, and B6R of MPXV were expressed and purified from a prokaryotic system. The four MPXV antigen proteins in combination were mixed with aluminum hydroxide or CpG7909 as adjuvant, and subsequently used to inoculate mice. The results of enzyme-linked immunosorbent assay (ELISA), flow cytometry analyses, and enzyme-linked immunospot (ELISPOT) assays indicated that A29L, M1R, A35R, and B6R elicited high-level antigen-specific antibodies and CD4^+^ T cells-based cellular immune response in mice. Moreover, the results of virus neutralization assays suggested that sera from the mice immunized with four proteins elicited high neutralizing activities against the vaccinia virus. Notably, the results of ELISA, ELISPOT, and virus neutralization assays also showed that the CpG7909 adjuvant was more effective in inducing an immune response compared with the aluminum adjuvant. In summary, this study offers valuable insights for further studies of subunit vaccine candidates for the prevention of MPXV and other orthomyxoviruses.

## 1. Introduction

Monkeypox (Mpox) is a zoonotic disease that sporadically occurs in Central and West African countries since the first human infection was reported in 1970 in the Democratic Republic of the Congo [1]. Since May 2022, Mpox has extended its epidemic magnitude and geographical reach. As a result, Mpox has affected 112 countries worldwide with 88,144 cases, including 149 deaths, as of 4 July 2023 [2]. The Mpox virus (MPXV) is a causative agent of Mpox and belongs to the genus *Orthopoxvirus* within the family *Poxviridae*, which also includes variola virus (VARV), cowpox virus (CPXV), vaccinia virus (VACV), and rabbitpox virus (RPXV) [3]. MPXV is an enveloped double-stranded DNA virus with a genome of approximately 197 kb in length. It encodes about 190 proteins, most of which are highly conserved among the members of the *Orthopoxvirus* genus [3,4].

Previous studies have suggested that antigens from *Orthopoxvirus* genus member viruses exhibited cross-protective immunity, and the smallpox vaccine provided 85% effectiveness against MPXV [5]. Two smallpox vaccines (ACAM2000 and JYNNEOS) have been approved by the U.S. Food and Drug Administration (FDA) for the emergent immunization of individuals at high risk of exposure and postexposure [6,7]. However, potential safety concerns, reports of reinfection, and breakthrough infection cases make these two smallpox vaccines far from the medical demand worldwide [8,9,10,11,12,13,14]. Therefore, a safe, effective, and widely applicable vaccine for MPXV prevention is needed.

Subunit vaccines, which offer advantages, including rapid development, high safety, and scalable industrial production, have emerged as a research hotspot and a promising direction for new vaccine development in preventing infectious diseases. The development of subunit vaccines against MPXV appears to be an attractive strategy, yet the rational selection of protective antigens is essential. Infective variants of *Orthopoxvirus* encompass the intracellular mature virions (IMVs) and the extracellular enveloped virions (EEVs) [15]. Protective antigens of VACV have been well characterized, including A27L, L1R, H3L, and D8L (corresponding to MPXV’s A29L, M1R, H3L, and E8L) of IMVs, while the effective targets on EEVs are limited to A33R and B5R (corresponding to MPXV’s A35R and B6R) [4,16,17]. Thus, an ideal MPXV subunit vaccine should incorporate antigens for both infectious forms. Previous studies showed that a DNA or protein-based smallpox vaccine, based on A27L, L1R, A33R, and B5R of VACV, could effectively protect non-human primates from a lethal MPXV challenge [18,19], and the DNA vaccine could also protect rabbits against lethal rabbitpox virus aerosol challenge [20]. These studies indicated that the use of these four antigen proteins or their homologous proteins of *Orthopoxvirus* for vaccine development might provide enough protection against the infection of corresponding viruses.

Therefore, in the present study, MPXV antigen proteins A29L, M1R, A35R, and B6R were modified, expressed, and purified from prokaryotic cells. These proteins either combined with aluminum hydroxide, or cytosine–phosphate–guanine 7909 (CpG7909) was used to immunize mice. The results of immune response analyses showed that the four proteins could induce high-level antigen-specific antibodies and CD4^+^ T cells-based cellular immune response in mice. Sera from the four proteins-immunized mice possessed high neutralizing activities against the vaccinia virus. In summary, the present study provides significant insights for further guidance for developing subunit vaccine candidates aimed at preventing MPXV and other orthomyxoviruses.

## 2. Materials and Methods

### 2.1. Cells, Virus, and Mice

Baby hamster kidney (BHK-21) cells (ATCC, #CCL-10) were cultured in Dulbecco’s modified Eagle’s medium (DMEM) (Corning, #10-013-CV), supplemented with 10% heat-inactivated fetal bovine serum (FBS) (Gibco, #10099141), 2 mM l-glutamine and antibiotics (100 μg/mL streptomycin and 100 U/mL penicillin), and incubated at 37 °C, 5% CO_2_ atmosphere with 95% relative humidity. VACV Tian Tan strain (GenBank No. AF095689) expressing a EGFP reporter gene (VACV-EGFP) was grown on BHK-21 cells [21]. Six-weeks-old specific pathogen-free female BALB/c mice (20–30 g) were purchased from Kangde Biological Co., Ltd. (Guangzhou, China). All animal experiments and research complied with the guidelines of the Institutional Animal Care and Use Committee, and the research has been ethically approved by the Laboratory Animal Center of Southern University of Science and Technology.

### 2.2. Protein Expression and Purification

The coding sequences of MPXV (GenBank No. ON563414) A29L (amino acids 1–110), M1R (amino acids 1–181), B6R (amino acids 20–279), and A35R (amino acids 58–181) were, respectively, added with the His-tag at C terminal, synthesized by GenScript (Nanjing, China), and cloned into pET-9a plasmid between Nde Ⅰ and BamH Ⅰ. The constructs were designated as pET-9a-A29L, pET-9a-M1R, pET-9a-B6R, and pET-9a-A35R, and used to transform E. coli BL21 (DE3) competent cells. A single clone of each transformant was picked to culture in LB medium containing 50 ng/mL kanamycin and shaken at 160 rpm. When the OD600 was about 0.8, pET-9a-A29L- or pET-9a-B6R-transformed cells were induced with 1 mM isopropyl b-D-1-thiogalactoside (IPTG) for 6 h at 25 °C, and pET-9a-M1R- or pET-9a-A35R-transformed cells were induced with 0.5 mM IPTG for 4 h at 37 °C.

For purification of A29L and B6R, the cells were first collected, resuspended in equilibrium buffer (50 mmol/L Tris-HCI, 500 mmol/L NaCl, 10 mmol/L imidazole, 10% glycerol, pH 7.4) and crushed using a high-pressure homogenizer (ATS, Los Angeles, CA, USA). After centrifuging at 4 °C 12,000× *g* for 15 min, A29L or B6R in the supernatant was isolated from the lysate, and loaded onto Ni-NTA affinity chromatography (GE, Boston, MA, USA). Then, proteins were eluted with elution buffer (50 mmol/L Tris, 500 mmol/L NaCl, 500 mmol/L imidazole pH 7.4). Endotoxin and imidazole were removed using size-exclusion chromatography (SEC, Cytiva, Washington, DC, USA). Then, the proteins were concentrated using an ultrafiltration centrifugal tube (Amicon Ultra filters; Millipore, MA, USA), collected in 0.01 M PBS, and stored at −20 °C for half a month.

For purification of M1R and A35R, the cells were collected, resuspended, crushed, and centrifugated as described above. After centrifugation, the insoluble fraction pellets, containing M1R or A35R, were washed three times with washing buffer (50 mmol/L Tris, 500 mmol/L NaCl, 2 mol/L urea, 1% Triton X-100, pH 7.4), dissolved in denaturing buffer (50 mmol/L Tris, 500 mmol/L NaCl, 8 mol/L urea, 50 mmol/L β-mercaptoethanol, pH 7.4), and centrifugated at 4 °C 12,000× *g* for 15 min. Then, the supernatant was collected and loaded onto Ni-NTA affinity chromatography. The renaturation of recombinant proteins in the nickel column was achieved by washing with gradient-reduced denature buffer. The NTA-Ni affinity chromatography and the size-exclusion chromatography were reported to eliminate the endotoxin effectively [22,23,24]. Finally, the elution, concentration, and storage of recombinant proteins were performed in the same way as described above.

### 2.3. Western Blotting (WB)

Fifty micrograms of each purified protein were loaded, separated by 12% SDS-PAGE, and transferred to PVDF membranes. Five percent of skim milk in 1 × TBST (#P1033, Solarbio, Beijing, China) was used to block the transferred membranes for 1 h at room temperature. Then, the membranes were incubated with the mouse anti-His antibodies (1:1000, #AF5060, Beyotime Biotech, Shanghai, China) for 12 h at 4 °C and incubated with the HRP-conjugated goat anti-mouse IgG antibody (1:1000, #A0216, Beyotime Biotech). After washing with TBST buffer (#P1033, Solarbio, Beijing, China), the target proteins were detected using BeyoECL Moon chemiluminescence reagent (Beyotime Biotech, #P0018FS) in a Tanon 5200 chemiluminescent imaging system.

### 2.4. Mice Immunization

Forty female BALB/c mice (6–8 weeks old) were divided into 5 groups (eight per group), respectively, designated as alum, AMBA/alum, AMBA, AMBA/CpG, and CpG. In the AMBA group, each mouse was intramuscularly immunized with a 40 μg mixture of A29, M1R, B6R, and A35R (10 μg each). In the AMBA/alum group, the inoculum was the same as AMBA group, but contained 40 μg alum adjuvant (Imject Alum Adjuvant, #77161, Thermo Scientific, Waltham, MA, USA). In the AMBA/CpG group, the inoculum was the same as AMBA group but contained 10 μg CpG adjuvant (CpG7909, #tlrl-2006-1, invivoGen, Toulouse, France). In the alum or CpG group, each mouse was, respectively, immunized with 40 μg alum adjuvant or 10 μg CpG adjuvant as control. For the boost immunization, mice in each group were immunized 14 days after the first immunization. Serum samples were collected from the orbital venous plexus on days 0, 14, 28, and 90, and stored at −80 °C for subsequent assays within 3 days.

### 2.5. Enzyme-Linked Immunosorbent Assay (ELISA)

The 96-well ELISA plates were coated with 200 ng expressed protein, incubated at 4 °C overnight, and washed 3 times using PBST (0.01 M PBS contains 0.05% Tween 20). Then, 100 μL 1% BSA was added to each well and blocked at 37 °C for 2 h. After washing 3 times with PBST, 100 μL serially diluted mouse sera were added into each well, followed by incubation for 2 h at room temperature. After washing 3 times with PBST, 100 μL HRP-conjugated anti-mouse IgG (1:10,000, #ab6789, Abcam, Cambridge, UK) was added to each well and incubated at 37 °C for 1 h. Finally, 100 μL TMB substrate (Beyotime, Shanghai, China) was added to each well and incubated for 20 min at room temperature. The absorbance was measured at 450 nm by Synergy HTX (BioTeK, Winooski, VT, USA) microplate reader.

### 2.6. Flow Cytometry Analysis

Two months after the second immunization, mice were euthanized to isolate splenocytes, and 2 × 10^6^ cells were inoculated in each well of a 24-well cell culture plate overnight. Ten micrograms of a mixture of A29L, M1R, B6R, and A35R (2.5 μg each) was added to the well for 6 h of stimulation, and 1 μL of Brefeldin A (1:1000, a protein transport inhibitor) was added for another 6 h of incubation. Then, cells were washed with 0.01 M PBS containing 0.2% BSA, blocked with the anti-CD16/CD32 antibody (1:100, #553142, BioLegend, San Diego, CA, USA), and stained with 100 μL FITC-conjugated anti-CD3 antibody (1: 100, BioLegend, San Diego, CA, USA), 100 μL Alexa Fluor 700-conjugated anti-CD4 antibody (1: 100, BioLegend, San Diego, CA, USA), and 100 μL APC/Fire 750-conjugated anti-CD8a antibody (1:100, BioLegend, San Diego, CA, USA) for half an hour. Then, the cells were washed with 0.01 M PBS containing 0.2% BSA, followed by intracellular cytokine staining. The cells were fixed, permeabilized with Fixation/Permeabilization Kit (#554714, BD Biosciences, San Diego, CA, USA), and stained with 100 μL Brilliant Violet 650-conjugated anti-IFN-γ (1: 100, BioLegend, San Diego, CA, USA), 100 μL Brilliant Violet 421-conjugated anti-IL-4 antibody (1: 100, BioLegend, San Diego, CA, USA), and 100 μL APC-conjugated anti-TNF-α (1:100, BioLegend, San Diego, CA, USA) antibodies at 4 °C for 30 min in the dark. Finally, after washing and resuspending with 0.01 M PBS with 2% BSA, the cells were analyzed using a multicolor flow cytometer (BD Biosciences), and data were analyzed with FlowJo software (version 10.8.1).

### 2.7. Enzyme-Linked Immunospot (ELISPOT) Assay

IFN-γ-secreting T cells were analyzed using a mouse IFN-γ ELISpot PLUS kit (2210005, Dakewe, Shenzhen, China) according to the manufacturer’s instructions. Briefly, the splenocytes suspension was obtained as described above, and 2 × 10^5^ cells were inoculated in each well of the 96-well plate and stimulated for 24 h as described above. The unstimulated splenocytes or phorbol-12-myristate-13-acetate (PMA) plus ionomycin (#2210005, Dakewe, Shenzhen, China)-stimulated splenocytes were, respectively, included as negative and positive controls. Deionized water was added to the cells and incubated at 4 °C for 10 min to lyse the cells. After washing six times with washing buffer, wells were added with 100 μL biotinylated anti-IFNγ antibody (1:100), and incubated at room temperature for 2 h. After washing six times, 100 μL streptavidin–HRP-conjugated secondary antibody (1:100) was added to each well and they were incubated at room temperature for 1 h. Then, wells were washed five times with washing buffer, and the residual liquid was discarded. AEC working solution was added to the well and incubated at 37 °C for 5–10 min. Finally, the AEC working solution was abandoned, and wells were washed with deionized water to stop the reaction. IFN-γ spots were counted using the AID ELISPOT Reader System and presented as the number of IFN-γ-secreting cells per 2 × 10^5^ splenocytes.

### 2.8. Virus Neutralization

Sera were heat-treated for 30 min at 56 °C to remove complement and other potential neutralizing agents prior to the infectious virus neutralization assay. Three-fold serially diluted sera, from 1:10 to 1:2430, were incubated with VACV-EGFP at a multiplicity of infection (MOI) of 1, for 1 h at 37 °C. Medium served as a negative control. Mice complement was added to the mixtures at a final concentration of 2%. The mixture was subsequently incubated with BHK21 cells for 1 h for adsorption. Then, the supernatants were replaced with fresh DMEM supplemented with 2% FBS. At 72 h post-infection, cells were fixed in 4% polyformaldehyde for 15 min followed by staining in DAPI for 5 min. Images were taken using an Operetta under High Content Screening (Perkin Elmer, Waltham, MA, USA), and infected cells were determined using Harmony Software version 4.6 (Perkin Elmer, Waltham, MA, USA). Serum dilution that completely inactivated VACV-EGFP with no fluorescence signal in cells was designated as neutralizing antibody titer.

### 2.9. Statistical Analysis

All data were analyzed with GraphPad Prism version 8.0 software. For all of the analyses, data are presented as mean ± standard errors in all experiments. *p* values were determined through one-way ANOVA or *t*-test, and a *p* value < 0.05 was considered to be statistically significant.

## 3. Results

### 3.1. Expression, Purification, and Characterization of Recombinant MPXV A29L, M1R, B6R, and A35R

**Figure 1:**
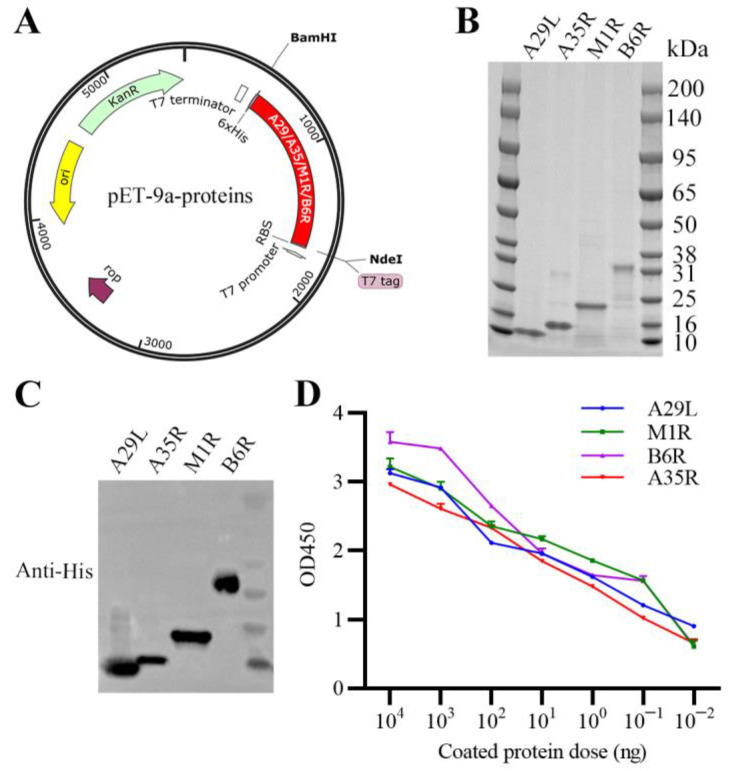
Purification and characterization of MPXV A29L, M1R, B6R, and A35R. (**A**) Construction of prokaryotic vectors expressing MPXV A29L, A35R, M1R, or B6R. The coding sequences of MPXV A29L, M1R, B6R, and A35R were transmembrane/cytoplasmic tails deleted, added with the His-tag at C terminal, and cloned into pET-9a plasmid, respectively. (**B**) Identification of purified A29L, A35R, M1R, and B6R of MPXV via SDS-PAGE. (**C**) Activity of purified MPXV A29L, A35R, M1R, and B6R was analyzed via WB assays. (**D**) Activity of purified MPXV A29L, A35R, M1R, and B6R was analyzed via ELISA assays. Data are presented as means ± SEM (*n* = 3).

The coding sequences of His-tagged recombinant A29L, M1R, B6R, or A35R were, respectively, inserted into pET-9a plasmid (Figure 1A). The expected sizes of purified recombinant proteins were 14 kDa (A29L), 15 kDa (A35R), 21 kDa (M1R), and 31 kDa (B6R), which were confirmed through SDS-PAGE identification (Figure 1B). The purified recombinant proteins were further characterized by WB and ELISA assays, and results showed that these proteins maintain optimal activities (Figure 1C,D). In addition, measured with a ToxinSensor Gel Clot Endotoxin Assay Kit (#L00351, GenScript, Nanjing, China), the endotoxin levels of these proteins were less than 0.25 EU per 10 μg, which is far below the permissible limit for immunization. These results suggested that MPXV antigen proteins A29L, M1R, B6R, and A35R were all successfully purified, and were suitable for subsequent vaccination.

### 3.2. Immunization with A29L, M1R, B6R, and A35R Elicited High-Level Antigen-Specific Antibodies in Mice

**Figure 2:**
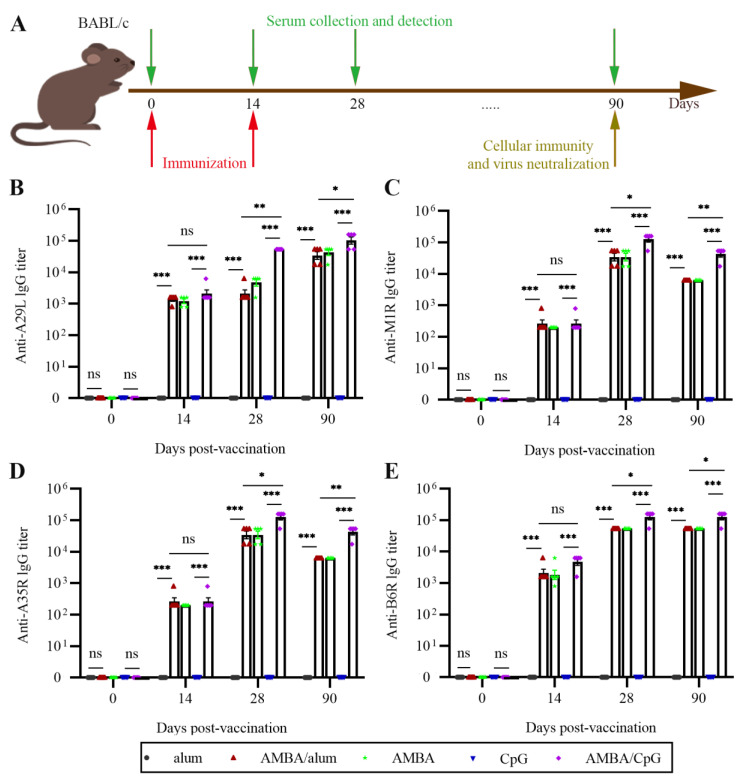
Antigen-specific antibody titers in mice immunized with the mixture of A29L, M1R, B6R, and A35R of MPXV. A29L, M1R, B6R, and A35R in combination were formulated with or without adjuvant and used to immunize mice intramuscularly. Sera of mice were sampled at days 0, 14, 28, and 90. Antigen-specific IgG antibody titers were evaluated via ELISA assays. (**A**) Schematic diagram of immunization and sera collection. (**B**) Anti-A27L antibody titers. (**C**) Anti-M1R antibody titers. (**D**) Anti-A35R antibody titers. (**E**) Anti-B6R antibody titers. Data are presented as means ± SEM (*n* = 3). *, *p* < 0.05; **, *p* < 0.01; ***, *p* < 0.001; ns, no significance (*p* > 0.05).

To evaluate the possibility of using recombinant A29L, M1R, B6R, and A35R as subunit vaccine, mice were inoculated intramuscularly twice, at 14-day intervals, with a combination of A29L, M1R, B6R, and A35R. Aluminum hydroxide or CpG7909 was used as an adjuvant, and mice in control groups were immunized with aluminum hydroxide or CpG7909 only. The inoculation regimen and collection of serum samples are shown in Figure 2A. Antigen-specific antibodies were detected via indirect ELISA assay. As shown in Figure 2B–D, after twice immunization, antibodies against all four target antigens were detected in mice from AMBA, AMBA/alum, and AMBA/CpG groups, but were not detected in mice from control groups. Although the antigen-specific IgG titers were slightly increased after the initial immunization, the IgG titers increased dramatically after the second immunization and remained at a high level two months later (Figure 2B–D). Importantly, antigen-specific IgG titers in the AMBA/CpG group were higher than those of AMBA and AMBA/alum groups (Figure 2B–D), which indicated that CpG7909 adjuvant provided a more homogeneous and stronger IgG antibody response. In the AMBA group, the anti-B6R and anti-A29L antibodies were sustained longer compared to anti-M1R and anti-A35R antibodies. These results showed that immunization with A29L, M1R, B6R, and A35R could effectively induce humoral immune response in mice.

### 3.3. Immunization with A29L, M1R, B6R, and A35R Elicited Cellular Immune Response in Mice

**Figure 3:**
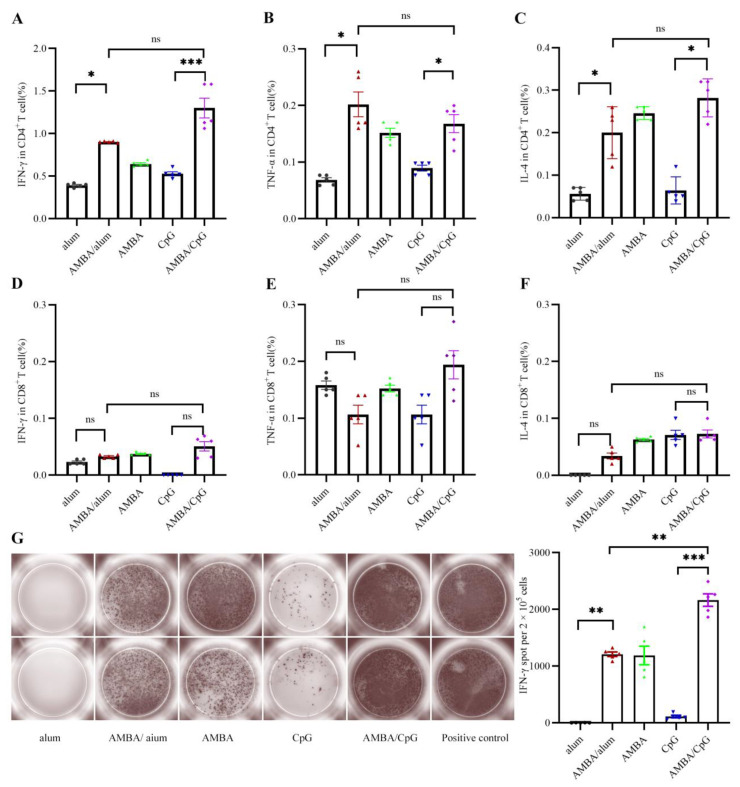
Cellular response in BALB/c mice immunized with the mixture of A29L, M1R, B6R, and A35R of MPXV. A29L, M1R, B6R, and A35R in combination were formulated with or without adjuvant and used to immunize mice intramuscularly. At day 90, splenocytes were isolated and stimulated a with mixture of A29L, M1R, B6R, and A35R, and T cell immune response was analyzed via flow cytometry analysis and ELISPOT assays. (**A**–**C**) IFN-γ, TNF-α, and IL-4 of CD4^+^ T cells were analyzed via flow cytometry assay. (**D**–**F**) IFN-γ, TNF-α, and IL-4 of CD8^+^ T cells were analyzed via flow cytometry assay. (**G**) IFN -γ-producing T cells were analyzed via ELISPOT assays. Data are presented as means ± SEM (*n* = 3). *, *p* < 0.05; **, *p* < 0.01; ***, *p* < 0.001; ns, no significance (*p* > 0.05).

T cell immune response induced by immunization with recombinant MPXV A29L, M1R, B6R, and A35R was detected via flow cytometry analysis and ELISPOT assays. Mice spleens were sampled two months after the second immunization. Splenocytes in the spleens were isolated, collected, and stimulated with a mixture of A29L, M1R, B6R, and A35R for the evaluation of the cellular immune responses. The immune phenotype of cellular response for type 1 helper T (Th1) cells cytokines (IFN-γ and TNF-α) and Th2 cell cytokines (IL-4) were detected. Results of flow cytometry analyses showed that antigen-specific CD4^+^ T cells expressing IFN-γ, TNF-α, and IL-4 were significantly elicited in AMBA, AMBA/alum, and AMBA/CpG groups, compared with those of CpG or alum control groups (Figure 3A–C). The antigen-specific CD8^+^ T cell response was detected to have no significant differences in any of the immunized groups (Figure 3D–F). These results indicated that the prokaryotic expressed recombinant MPXV A29L, M1R, B6R, and A35R were mainly processed and presented through the class II major histocompatibility complex (MHC-II) molecular pathway.

The cellular immune response was further evaluated via ELISPOT assay. As shown in Figure 3G, the frequencies of IFN-γ-secreting splenocytes in AMBA/alum, AMBA, and AMBA/CpG groups were significantly higher than those of alum or CpG group which were shown at background levels. Meanwhile, splenocytes of mice in the AMBA/CpG group exhibited much higher frequencies of IFN-γ secreting than those of mice in AMBA/alum and AMBA groups. These results suggested that immunization with A29L, M1R, B6R, and A35R could induce CD4^+^ T cell-based cellular immune response in mice.

### 3.4. Immunization with MPXV A29L, M1R, B6R, and A35R Elicited Neutralizing Antibodies against VACV

**Figure 4:**
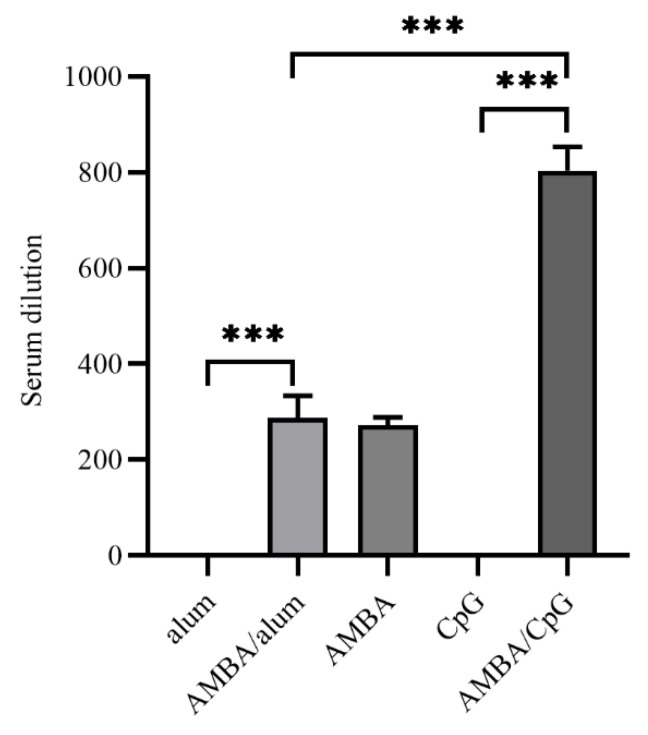
Antibody titers of neutralizing antibodies against VACV in mice immunized with A29L, M1R, B6R, and A35R of MPXV. Neutralizing antibody levels against VACV were determined via virus neutralization assays. Serum samples were heat-treated to remove complement and other potential neutralizing agents, three-fold serially diluted from 1:10 to 1:2430, incubated with VACV-EGFP, and used to infect BHK21 cells. Serum dilution that completely inactivated VACV-EGFP with no fluorescence signal in cells was designated as neutralizing antibody titer. Data are presented as means ± SEM (*n* = 3). ***, *p* < 0.001.

Two months after the second immunization, the antiviral neutralizing activities of sera from immunized mice were determined through a virus neutralization assay using VACV, which also belongs to the same genus as MPXV. Results demonstrated that sera from mice that only immunized alum or CpG adjuvant in control groups had no neutralizing activities against VACV, whereas mice in AMBA, AMBA/alum, as well as AMBA/CpG groups developed neutralizing antibodies against VACV (Figure 4). In the condition that VACV was completely inactivated by a neutralizing antibody, the neutralizing antibody titers of AMBA and AMBA/alum groups were both 1: 270, while the neutralizing antibody titer of the AMBA/CpG group was 1: 810, indicating that CpG7909 adjuvant was more effective in facilitating antigen-induced neutralizing antibodies.

## 4. Discussion

Although the World Health Organization (WHO) officially ended the 10-month-long global health emergency for MPXV on May 11, 2023, “there is a need to move to a strategy for managing the long-term public health risks of MPXV than to rely on emergency measures,” as stated by the Vice Chair of the WHO’s emergency committee on MPXV. This transition necessitates the development of safe and effective vaccines to support the implementation of this strategy. Considering the instances of reinfection and breakthrough infection of MPXV observed in vaccinated individuals [8,9,10,11,12,13], along with potential safety concerns [14] and the risk of inadequate effectiveness and relatively low levels of neutralizing antibodies against MPXV induced by JYNNEOS and ACAM2000 [25], it becomes obvious that the safeguarding provided by smallpox vaccines and natural immunity might sometimes fail in ensuring protection. Therefore, MPXV vaccines should prioritize specific and defined targets rather than relying on attenuated viruses.

The successful application of messenger RNA (mRNA) vaccines during the coronavirus disease 2019 (COVID-19) pandemic has showcased their efficacy. This technology could be a promising direction for the development of MPXV vaccine. Currently, several mRNA vaccines against MPXV are being developed, demonstrating promising results in inducing humoral and cellular immune responses. These vaccines also show effectiveness against VACV in mouse models [14,26,27,28,29,30,31]. Apart from mRNA technology, another promising avenue for vaccine development is subunit vaccines. For example, the RBD-Dimer-based Covid-19 Vaccine ZF2001 developed by Anhui Zhifei Longcom Biopharmaceutical (Hefei, China) was an efficient subunit vaccine against COVID-19 [32]. Furthermore, several other subunit vaccines have been evaluated by Chinese authorities for inclusion in emergency use and selected as a recommended vaccine in the Implementation Plan for the Second Dose of the Novel Coronavirus Vaccine.

In the current study, we expressed MPXV antigen proteins A29L, M1R, B6R, and A35R in the prokaryotic system. These four proteins were mixed in combination with the aluminum hydroxide adjuvant or CpG7909 adjuvant and were used to immunize mice for evaluation as subunit vaccine candidates against MPXV. Aluminum hydroxide and CpG790 were used as adjuvant because alum adjuvant is the most widely used vaccine adjuvant at present [33], and CpG oligodeoxynucleotide is a novel biological adjuvant which is effective in stimulating immune response with great research value [33,34]. In addition, ongoing clinical studies indicate that CpG7909 is safe and well tolerated and can improve vaccine-induced immune responses [35,36]. Results showed that whether formulated with or without adjuvant, the four antigen proteins could elicit a robust humoral immune response and CD4^+^ T cells-based cellular immune response (Figure 2, Figure 3 and Figure 4). In general, the CpG7909 adjuvant was more effective in allowing these four MPXV antigen proteins to elicit higher humoral and cellular immune responses compared with the aluminum adjuvant. In the AMBA group without adjuvant, the anti-B6R and anti-A29L antibodies maintained longer than anti-M1R and anti-A35R antibodies. This might be because A29L and B6R possessed better immunogenicity than M1R and A35R, and further studies are needed to investigate it. Interestingly, several MPXV antigen proteins-based mRNA vaccines were reported to induce cellular immune response activating both CD4^+^ and CD8^+^ T cells [14,28,29]. This is probably because mRNA was delivered into mouse cells, and the antigen proteins were expressed in eukaryotic cells with correct folding and post-translational modification such as glycosylation, which would contribute to activating CD8^+^ T cells. In this study, the recombinant A29L, M1R, B6R, and A35R proteins of MPXV were expressed in prokaryotic cells, which led to these proteins’ lack of post-translational modifications. Our results of WB and ELISA assays showed that these proteins maintain antigenicity well (Figure 1C,D); this might be because antigenicity of these proteins mainly focused on their linear epitopes. Nevertheless, the loss of conformational epitopes might result in these four proteins being processed and presented through an MHC-II molecular pathway, which can result in the activation of only CD4^+^ T cells in immunized mice. Even if only CD4^+^ T cells are induced, immunization with these four proteins should provide enough protection against MPXV, since B cells and immunoglobulins play critical roles in humoral immune response and were reported to be sufficient for protection against *Orthopoxvirus* [37,38,39,40]. Furthermore, mice with CD8^+^ T cell dysfunction were still able to be protected by vaccination as well [41]. Certainly, more research is needed to clarify the mechanisms and differences of cellular immune response induced by mRNA vaccine and subunit vaccine based on the same antigen proteins. Further studies are necessary and these should include viral challenges of non-human primates and humans.

In conclusion, we here report the development and evaluation of a subunit vaccine candidate composed of MPXV A29L, M1R, A35R, and B6R, which could elicit a strong immune response in mice. Our study provides useful information that can guide future research on subunit vaccine candidates aimed at preventing MPXV and other orthomyxoviruses.

## Data Availability

The data that support the findings of this study are available from the corresponding author upon reasonable request.
